# LDL-c/HDL-c Ratio and NADPH-Oxidase-2-Derived Oxidative Stress as Main Determinants of Microvascular Endothelial Function in Morbidly Obese Subjects

**DOI:** 10.3390/antiox13091139

**Published:** 2024-09-20

**Authors:** Jorge Santos, José M. La Fuente, Argentina Fernández, Paula Ruano, Javier Angulo

**Affiliations:** 1 Unidade de Cirurgia Esofagogástrica e Tratamento Cirúrgico de Obesidade, Centro Hospitalar e Universitário de Santo António (CHUdSA), 4099-001 Porto, Portugal; jorgenunessantos@sapo.pt; 2 Serviço de Urologia, Centro Hospitalar e Universitário de Santo António (CHUdSA), 4099-001 Porto, Portugal; lafuentecarvalho@gmail.com; 3 Servicio de Histología-Investigación. Unidad de Investigación Traslacional en Cardiología—IRYCIS/UFV, Hospital Universitario Ramón y Cajal, 28034 Madrid, Spain; argentina.fernandez@salud.madrid.org (A.F.); paularuanoaparicio@gmail.com (P.R.); 4 Centro de Investigación Biomédica en Red de Fragilidad y Envejecimiento Saludable (CIBERFES), Instituto de Salud Carlos III, 28029 Madrid, Spain

**Keywords:** endothelial dysfunction, human obesity, low-density lipoprotein cholesterol, high-density lipoprotein cholesterol, NADPH-oxidase, human mesenteric small arteries

## Abstract

The identification of obese subjects at higher risk for cardiovascular disease (CVD) is required. We aimed to characterize determinants of endothelial dysfunction, the initial step to CVD, in small omental arteries of visceral fat from obese subjects. The influences of analytical parameters and vascular oxidative stress mediated by NADPH-oxidase-2 (NOX2) on endothelial function were determined. Specimens were obtained from 51 obese subjects undergoing bariatric surgery and 14 non-obese subjects undergoing abdominal surgery. Obese subjects displayed reduced endothelial vasodilation to bradykinin (BK). Endothelial vasodilation (pEC_50_ for BK) among obese subjects was significantly and negatively associated with low-density lipoprotein cholesterol (LDL-c)/high-density lipoprotein cholesterol (HDL-c) ratio (r = -0.510, *p* = 0.0001) in both women and men, while other metabolic parameters and comorbidities failed to predict endothelial function. The vascular expression of NOX2 was upregulated in obese subjects and was related to decreased endothelial vasodilation (r = −0.529, *p* = 0.0006, *n* = 38) and increased oxidative stress (r = 0.783, *p* = 0.0044, *n* = 11) in arterial segments. High LDL-c/HDL-c (>2) and high NOX2 (above median) were independently associated with reduced endothelial function, but the presence of both conditions was related to a further impairment. Concomitant elevated LDL-c/HDL-c ratio and high vascular expression of NOX2 would exacerbate endothelial impairment in obesity and could reveal a deleterious profile for cardiovascular outcomes among obese subjects.

## 1. Introduction

Twenty-three percent of adults are living with obesity in the European Region, with levels even higher in some countries. In this region, obesity is more prevalent among females (24%) than among males (22%), in contrast to that observed with overweight. Mediterranean and eastern European countries display the highest levels of both overweight and obesity, with higher obesity prevalence in people with lower educational attainment [1]. In fact, the incidence of obesity is even growing after the COVID-19 pandemic [2]. Obesity is linked to increased risk for many noncommunicable diseases (NCDs) including cardiovascular diseases (CVDs), type 2 diabetes, several types of cancer and obstructive sleep apnea syndrome (SAOS). More than two-thirds of deaths related to high body mass index (BMI) were reported to be due to CVDs [3]. Considering the pandemic figures of obesity, the burden of CVDs related to this condition represents an outstanding challenge for healthcare systems.

However, obese populations are heterogeneous, and the risk for CVD in each obese subject could significantly differ. Thus, the identification of obese subjects at higher risk would be clinically relevant [4]. Endothelial dysfunction is considered an initial step in the track to CVDs including atherosclerosis [5]. Indeed, there is substantial evidence for the impact of obesity on endothelial function in animal models [6,7] and humans [8,9]. In this sense, lipid profile has been proposed to be determinant in the development of atherosclerosis and CVD [10]. Low-density lipoprotein cholesterol (LDL) has been shown to induce endothelial dysfunction [11], while increased triglyceride/high-density lipoprotein cholesterol (HDL) ratio has been related to endothelial dysfunction in obese children [12].

The impact of obesity on endothelial function has been mainly attributed to diminished nitric oxide (NO) bioavailability, leading to impaired endothelium-dependent vasodilation, but other mechanisms related to endothelium-derived vasoconstrictors such as prostanoids and endothelin-1 have also been suggested to participate [13]. Endothelium-derived hyperpolarization (EDH) is an important contributor to endothelial vasodilation in small resistance vessels [14]. The impairment of EDH-mediated responses was proposed to account for endothelial dysfunction in obesity [15,16], but other evidence in animal models suggested the upregulation of this vasodilating pathway in obesity as a compensatory mechanism [17,18]. Contradictory results could be related to a sex-specific effect [19].

At the vascular level, obesity shares similarities with aging pathogenetic mechanisms, both conditions being tightly related to inflammation and oxidative stress resulting in the accumulation of reactive oxygen species (ROS) [20,21]. An excess of ROS is contributed to by NADPH-oxidases (NOXs), especially NOX-2, which generates superoxide and reduces NO bioavailability [22], displaying a crucial role for endothelial dysfunction in middle-aged mice with diet-induced obesity [23]. NOX4 is an isoform also important in vascular tissue [24,25]. However, its role in vascular pathophysiology is complex since it has been related to vascular alterations [26,27] but also has been proposed to have a protective role in vascular pathology [28,29].

The aim of this work was to characterize the determinants of endothelial vasodilation in omental arteries of visceral fat from human obese subjects. The influences of conventional analytical parameters and vascular oxidative stress mediated by NOX2 and NOX4 on endothelial function were determined with the aim of revealing a potential risk profile among obese subjects.

## 2. Materials and Methods

### 2.1. Human Samples

Human omentum specimens were obtained from 51 patients with morbid obesity who underwent bariatric surgery in the Centro Hospitalar e Universitário de Santo António (CHUdSA) with mixed techniques combining Roux-en-Y gastric bypass and vertical sleeve gastrectomy. A group of 14 patients undergoing surgery for gastroesophageal reflux, hiatal hernia or cholelithiasis served as a control. Sample size was decided based on previous experience with similar approaches [30,31]. Visceral fat biopsies were collected without altering the established surgical protocol. Patients gave informed consent prior to surgery. Patients with active infectious diseases or unable to provide informed consent were excluded. Time from extraction to evaluation ranged between 16 and 24 h. During this time, the tissues were immersed in M-400 solution (concentration in g/100 mL: mannitol, 4.19; KH_2_PO_4_, 0.205; K_2_HPO_4_·3H_2_O, 0.97; KCl, 0.112; NaHCO_3_, 0.084; pH 7.4) and maintained at 4 °C to 6 °C until used [32]. Protocols and consent forms were approved by the Ethics Committee of CHUdSA in Porto, Portugal (248-20 (192-DEFI/196-CES), and all procedures followed the ethical principles of the Declaration of Helsinki for medical research involving human subjects.

### 2.2. Evaluation of Vascular Function in Isolated Human Small Arteries

Visceral fat specimens were placed in a petri dish containing cold Krebs–Henseleit solution (KHS; composition in mM: NaCl 115, CaCl_2_ 2.5, KCl 4.6, KH_2_PO_4_ 1.2, MgSO_4_·7H_2_O 1.2, NaHCO_3_ 25, glucose 11.1 and Na_2_EDTA 0.03) for dissection. Small mesenteric arteries (200–700 µm) were isolated by carefully removing the adhering fat. Arterial vascular segments (1.6–1.9 mm long) were subsequently mounted on microvascular wire myographs (Danish MyoTechnology; Aarhus, Denmark) for isometric tension recordings as previously described [30,31]. The arteries were allowed to equilibrate for 30 min in KHS at 37 °C, continuously bubbled with 95% O_2_/5% CO_2_ mixture to maintain a pH of 7.4. The internal circumference of vascular segments when relaxed in situ under a transmural pressure of 100 mmHg (L_100_) was determined. The vessels were then set to an internal circumference equivalent to 90% of L_100_, at which the force development was close to maximal. The arterial segments were then exposed to 125 mM K^+^ (KKHS; equimolar substitution of NaCl by KCl in KHS), and the contractile response was measured. Human mesenteric arterial (HMA) segments failing to produce a tension equivalent to a pressure of 100 mmHg were rejected. Contractile responses were evaluated by cumulative additions of the thromboxane analogue, U46619 (0.1 nM to 10 µM), or endothelin-1 (ET-1, 0.1 nM to 0.3 µM) to the chambers. For relaxation responses, arterial segments were contracted with U46619 (10–30 nM) or with KCl (25–35 mM), which produced approximately 80% of the maximum response to 125 mM K^+^. When the contraction reached a plateau, the endothelium-dependent relaxation was assessed by adding increasing concentrations of bradykinin (BK; 1 nM to 1 µM) to the organ bath. Some arterial segments contracted with U46619 were treated with N^G^-nitro-L-arginine methyl ester (L-NAME, 100 µM) and indomethacin (10 µM) to inhibit NO and prostacyclin production in order to assess BK-induced vasodilation mediated by endothelium-derived hyperpolarizing factor (EDHF). HMA segments contracted with KCl (which precludes a hyperpolarizing response) were treated with indomethacin (10 µM) to isolate BK-induced vasodilation mediated by NO. Responses to sodium nitroprusside (SNP; 1 nM to 10 μM) were used to evaluate non-endothelium-dependent relaxation.

### 2.3. Western Blot Analysis

Small mesenteric vascular tissue samples derived from control and obese subjects were dissected, rinsed in KHS to eliminate blood, frozen in liquid nitrogen and maintained at −80 °C until proteins were extracted. Total proteins were extracted from vascular tissue by homogenization in T-PER lysis buffer (Pierce Biotechnology, Inc., Rockford, IL, USA) according to the manufacturer’s instructions, and a Protease Inhibitor Cocktail (1×, Roche Diagnostics, IN, USA) was included. Protein content in homogenates was determined by the bicinchoninic acid (BCA) method. Western blot analyses were performed as previously described [32,33]. A total of 10 µg of protein extracts were separated by SDS-PAGE on a 10% polyacrylamide gel. Proteins were transferred to polyvinylidene difluoride membranes and blocked for 5 min with EveryBlot blocking buffer (Bio-Rad, Hercules, CA, USA). Membranes were incubated overnight at 4 °C with a specific rabbit antibody against NADPH-oxidase-2 (NOX2, gphox-21) (EpiGentek, Farmingdale, NY, USA, cat.# A70477, dilution 1:1000) or NOX4 (EpiGentek, cat.#A72493, dilution 1:750) and a mouse antibody against β-actin (Novus, cat.# NB600-501, dilution 1:5000), which was used as loading control. Consequently, membranes were incubated with goat anti-rabbit (1:10,000 dilution; Novus, cat.# NB7160) or goat anti-mouse (1:5000 dilution; Novus, cat.# NBP2-30347H) horseradish peroxidase-conjugated secondary antibody for 1 h at room temperature. Blots were visualized by the ECL detection system (ThermoFisher Scientific, Waltham, MA, USA). Results were quantified by densitometry, using QuantityOne/Chemi-Doc Image-Lab 6.0 Software (Bio-Rad). Data were expressed as the ratio of band intensity for NOX2 or NOX4 with respect to respective β-actin band intensity.

### 2.4. Immunofluorescence Assays

Segments of human mesenteric small arteries (3–4 mm in length) were dissected and immersed in saccharose (30% *w*/*v*), embedded in OCT and stored at −80 °C until immunofluorescence assays were performed. Then, OCT blocks were cut into 6 μm thick transversal sections with a cryostat and mounted on polylysine-coated glass slides. For the immunodetection of NOX2, OCT was removed and sections were treated with acetone and methanol for fixing the tissues and removing the autofluorescence. Sections were incubated with rabbit antibodies against NOX2 (EpiGentek cat.# A70477, 1:100 dilution) overnight at 4 °C. After washout in phosphate-buffered saline plus 0.3% Triton X-100, the sections were incubated with a secondary Alexa Fluor 488-conjugated goat anti-rabbit antibody (dilution 1:250; Life Technologies, Alcobendas, Spain) and with diamidino-2-phenylindole (DAPI; Life Technologies) to counterstain the nuclei for 1 h at room temperature. The sections were mounted and viewed using fluorescence microscopy (Olympus BX51, Olympus Corporation, Tokyo, Japan).

In situ reactive oxygen species (ROS) production was measured using the fluorescent dye dihydroethidium (DHE) as described previously [34]. Briefly, sections of human mesenteric arterial segments were fixed with cold acetone, cleaned from OCT and incubated with DHE (4 μM; Invitrogen, Life Technologies Corporation, Eugene, OR, USA) for 30 min at 37 °C in a humidified chamber protected from light. In the presence of ROS, DHE is oxidized to ethidium, which yields bright red fluorescence. Nuclei were counterstained with 300 nM diamidino-2-phenylindole (DAPI, Life Technologies). After washing with PBS plus 0.05% Triton X-100, sections were mounted and visualized by fluorescence microscopy (Olympus BX51, Olympus Corporation, Tokyo, Japan). Random images (3 to 5 fields at 400× magnification) from each specimen were captured, and the ratio of red fluorescence intensity (DHE) normalized to the number of nuclei was determined with Image J 1.48i imaging software (McBiophotonics Image J, NIH, Bethesda, MD, USA). The same background threshold was subtracted from fluorescence quantification for all images. An average value for each specimen was obtained. Characteristics of the specimen were blinded for the investigator capturing and quantifying immunofluorescence images.

### 2.5. Biochemical Measurements

Blood samples were collected from study subjects for the measurement of serum fasting glucose, glycosylated hemoglobin (HbA_1C_), serum fasting insulin, serum lipid profile and transaminases following routine procedures of the Laboratory of Clinical Analyses of CHUdSA or University Hospital Center of Santo António. HOMA-IR was calculated as described by Mathews et al. [35].

### 2.6. Data Analysis

Continuous variables were expressed as mean ± SEM and compared by the Mann–Whitney U-test, while categorical variables were analyzed by Fisher’s exact test. Kruskal–Wallis followed by Dunn’s test was used for multiple comparisons. For a comparison of complete concentration–response curves, a two-factor analysis of variance (ANOVA) test was applied (one factor the vasoactive agent and the other the specific condition). When multiple curves were compared, Bonferroni’s correction was performed. pEC_50_ is defined as the –log M of the concentration required for obtaining 50% relaxation (BK, SNP) or 50% of K^+^-induced contraction (U46619, ET-1). E_max_ is defined as the maximum response (relaxation or contraction). The number of subjects used for determinations is indicated in each graph. Models of linear regression (Pearson correlation) were constructed with the pEC_50_ or E_max_ considered as the dependent variable. In all cases, a probability value of less than 5% was considered significant. Data were analyzed using GraphPad Prism software (6.0 version, San Diego, CA, USA).

## 3. Results

### 3.1. Characteristics of Study Subjects

Table 1 shows the clinical characteristics of patients included as controls (n = 14) as well as those from obese subjects (*n* = 51). In addition to an obvious increase in weight and body mass index (BMI), obese subjects were significantly younger and more frequently presented diabetes, sleep apnea and liver steatosis while displaying significantly higher levels of insulin and HOMA-IR. Other parameters were not significantly different between both populations.

### 3.2. Impact of Obesity on Vascular Function

**Figure 1:**
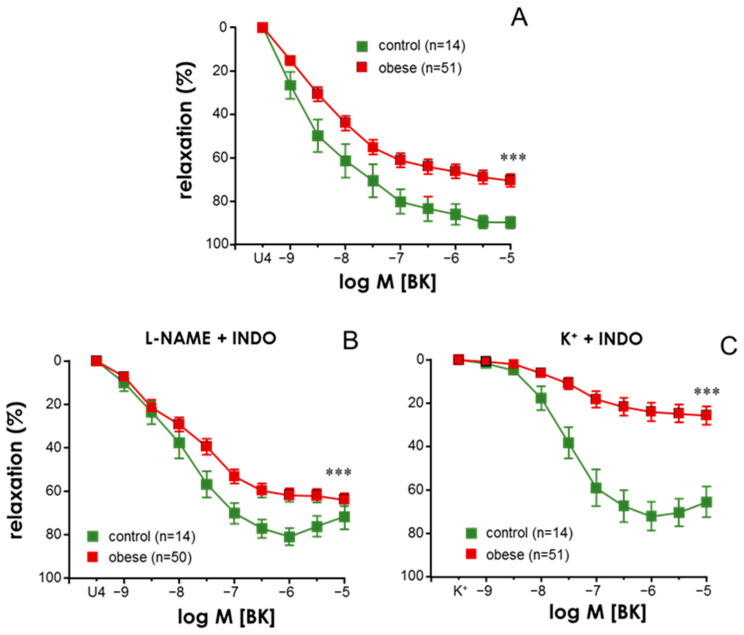
**Obesity is related to the impairment of endothelium-dependent vasodilation through endothelial hyperpolarization and nitric oxide-mediated relaxation in human mesenteric small arteries.** Endothelium-dependent vasodilations induced by bradykinin (BK, 1 nM to 10 µM) in human mesenteric arteries (HMAs) contracted with the thromboxane analogue, U46619 (10–30 nM) (**A**,**B**), or 25–35 mM K^+^ © obtained from non-obese subjects (control, BMI < 30) and from subjects with morbid obesity (obese, BMI > 35). Panel (**A**) shows BK-induced responses in HMAs without treatment, while HMAs were treated with indomethacin (INDO, 10 µM) plus N^G^-nitro-L-arginine methyl ester (L-NAME, 100 µM) in panel (**B**) and with INDO (10 µM) in panel (**C**). Data are expressed as the mean ± S.E.M. of the percentage of relaxation. *n* indicates the number of patients from whom the vessels were collected. *** indicates *p* < 0.001 vs. control by a two-factor ANOVA test.

No significant differences in the diameter of mesenteric vascular segments were observed between those obtained from control subjects (486.9 ± 26.6 µm, *n* = 54) and those from obese subjects (511.3 ± 46.9 µm, *n* = 169). In the same way, contractions induced by 125 mM K^+^ in segments from control subjects (13.91 ± 1.03 mN) were not significantly different from those in vessels from obese subjects (15.13 ± 0.52 mN). However, with respect to responses obtained in arteries from control subjects, endothelium-dependent vasodilation was significantly impaired in mesenteric microvessels from obese subjects contracted with the thromboxane analogue, U46619 (10–30 nM) (Figure 1A). This impairment was also detected in arterial segments treated with the NO synthase (NOS) inhibitor, L-NAME (100 µM), and the cyclooxygenase (COX) inhibitor, indomethacin (10 µM) (Figure 1B), as well as in those segments contracted with 25–35 mM KCl and treated with indomethacin (Figure 1C). This means that the impairment in endothelial vasodilation involved defective responses mediated by both endothelium-derived hyperpolarization (EDH) and NO, respectively. However, the magnitude of the impairment seemed to be more pronounced with respect to endothelial NO-mediated vasodilation (Figure 1C). In contrast, the presence of obesity did not significantly modify endothelium-independent vasodilation induced by the NO-donor, sodium nitroprusside (SNP) (pEC_50_ 8.62 ± 0.11, *n* = 11, vs. 8.47 ± 0.09, *n* = 7, control vs. obese subjects, respectively, *p* = 0.3649).

### 3.3. Endothelial Function and Lipid Profile in Obesity

**Figure 2:**
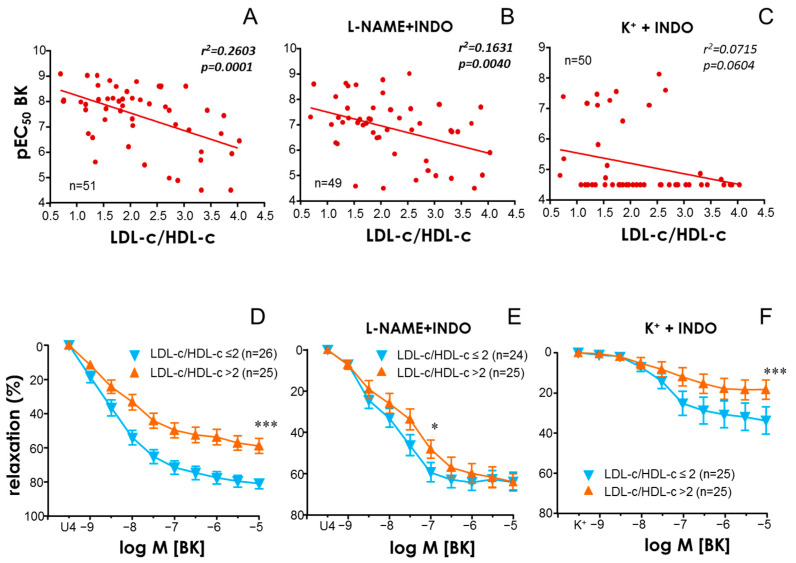
**Low-density lipoprotein cholesterol (LDL-c)/high-density lipoprotein cholesterol (HDL-c) ratio determines endothelial vasodilation in obese subjects.** Upper panels show linear regressions of LDL-c/HDL-c ratio versus pEC_50_ for bradykinin (BK) in subjects with morbid obesity (**A**–**C**). pEC_50_ values for BK for each subject were obtained in human mesenteric arteries (HMAs) contracted with the thromboxane analogue, U46619 (10–30 nM) (**A**,**B**), or 25–35 mM K^+^ (**C**). Lower panels show complete BK-induced vasodilations in HMAs contracted with the thromboxane analogue, U46619 (10–30 nM) (**D**,**E**), or 25–35 mM K^+^ (**F**) obtained from obese subjects displaying LDL-c/HDL-c ratios equal to or below 2 (LDL-c/HDL-c ≤ 2) versus those with LDL-c/HDL-c above 2 (LDL-c/HDL-c > 2). Data in panels (**A**,**D**) were obtained in HMAs without treatment, while HMAs were treated with indomethacin (INDO, 10 µM) plus N^G^-nitro-L-arginine methyl ester (L-NAME, 100 µM) in panels (**B**,**E**) and with INDO (10 µM) in panels (**C**,**F**). *n* indicates the number of patients from whom the determinations were obtained. Coefficients of determination and probability (*p*) values are indicated in upper panels. Significant associations are highlighted in bold plus italic. Data in lower panels are expressed as the mean ± S.E.M. of the percentage of relaxation. * indicates *p* < 0.05; *** *p* < 0.001 vs. control by a two-factor ANOVA test.

BK-induced responses in arteries from control subjects were not significantly associated with age or any lipid parameter but were significantly and negatively associated with HOMA-IR (Table 2). On the other hand, pEC_50_ for BK in vessels from obese subjects was significantly and inversely associated with serum concentrations of LDL-c (Table 2) as well as with the ratio LDL-c/HDL-c (Table 2, Figure 2A), this last parameter of lipid profile being the most closely associated with the decline in endothelial vasodilation to BK (*p* = 0.0001). No other parameter, including triglycerides, glucose, HbA_1C_, HOMA-IR or age, was significantly associated with endothelial vasodilation in arteries from obese subjects (Table 2). In the presence of NOS and COX inhibition, i.e., when EDH was in charge of vasodilation, pEC_50_ for BK was also significantly and inversely associated with LDL-c/HDL-c ratio (Figure 2B). However, LDL-c/HDL-c ratio was not significantly associated with the pEC_50_ for BK when NO component was isolated, i.e., in arteries contracted with KCl and treated with COX inhibitor. Since, under these conditions, maximum relaxation (E_max_) in arteries from a number of obese subjects did not reach 50% and pEC_50_ was estimated to be below 5, the linear regression of E_max_ to BK versus the LDL-c/HDL-c ratio in arteries contracted with KCl and treated with indomethacin was constructed, but, despite the existence of a trend, no significant association was reached (r = −0.2604, *p* = 0.0678). Supporting the relevance of the LDL-c/HDL-c ratio in the endothelial vasodilation of obese subjects, BK-induced relaxation in arteries from obese subjects with LDL-c/HDL-c ratios above 2 (median value) was significantly reduced with respect to subjects with lower LDL-c/HDL-c values (Figure 2D–F). In fact, this was true for any of the components of endothelial vasodilation, including responses involving only NO (Figure 2F).

On the other hand, contractile responses elicited by the thromboxane analogue, U46619, or by the vasoactive peptide, endothelin-1 (ET-1), in mesenteric microvessels from obese subjects were not significantly different to those obtained in arteries from control subjects (Supplementary Figure S1A,B). Nevertheless, LDL-c/HDL-c ratio, in addition to fit with endothelial function, was significantly and directly associated with maximum contraction (E_max_) induced by either U46619 or ET-1 in mesenteric vessels from obese subjects (Supplementary Figure S1C,D).

### 3.4. Impact of Comorbidities on Endothelial Function in Obese Subjects

The presence of diabetes, hypertension, liver steatosis or sleep apnea did not significantly influence endothelial vasodilation to BK in mesenteric microvessels from obese subjects. Table 3 compares parameters of BK-induced vasodilations (pEC_50_ and E_max_) between subjects with or without such comorbidities. No significant differences in these parameters were observed.

### 3.5. Clinical Characteristics Associated with Poor Endothelial Function

Trying to further define the profile of obese subjects displaying defective endothelial function, BK-induced responses represented by their pEC_50_ were divided into tertiles. The characteristics of 17 obese subjects in the lowest tertile of pEC_50_ values for BK (<7.29) in comparison to those subjects in the first and second tertiles are shown in Table 4. Consistently with the linear regression data, the only significant differences observed in these patients were decreased serum HDL-c and increased serum LDL-c, resulting in a significant increase in LDL-c/HDL-c ratio (Table 4). No other parameters including glucose metabolism or comorbid conditions were significantly different in these patients (Table 4). Moreover, despite the fact that BK-induced relaxation in arteries precontracted with high K^+^ and treated with indomethacin were not significantly related to LDL-c/HDL-c ratio in lineal regressions, obese subjects within the tertile of best vasodilation under these conditions (>30% relaxation) displayed a significantly decreased LDL-c/HDL-c ratio (Supplementary Table S1).

### 3.6. Gender Stratification of Association of LDL-c/HDL-c Ratio with Endothelial Function in Obese Subjects

**Figure 3:**
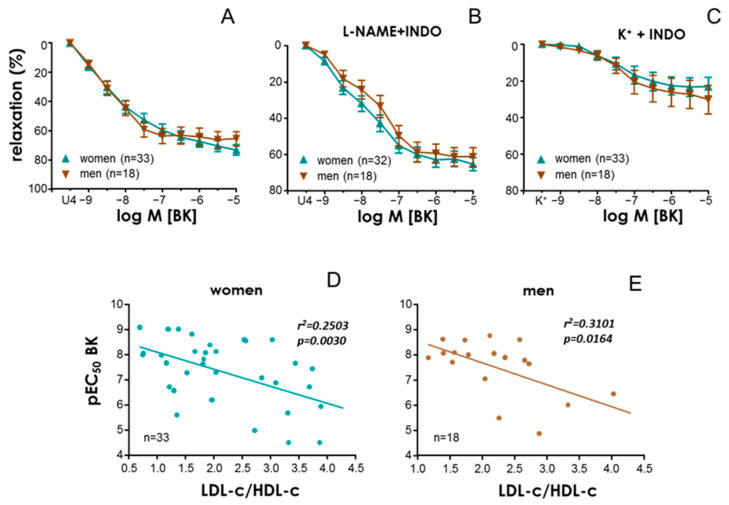
**A negative association of low-density lipoprotein cholesterol (LDL-c)/high-density lipoprotein cholesterol (HDL-c) ratio with endothelial vasodilation is observed in both female and male obese subjects.** Upper panels show vasodilations induced by bradykinin (BK, 1 nM to 10 µM) in HMAs contracted with the thromboxane analogue, U46619 (10–30 nM) (**A**,**B**), or 25–35 mM K+ (**C**) obtained from female (women) and male (men) obese subjects. Data in panel (**A**) were obtained in HMAs without treatment, while HMAs were treated with indomethacin (INDO, 10 µM) plus N^G^-nitro-L-arginine methyl ester (L-NAME, 100 µM) in panel (**B**) and with INDO (10 µM) in panel (**C**). Data are expressed as the mean ± S.E.M. of the percentage of relaxation. Lower panels show linear regressions of LDL-c/HDL-c ratio versus pEC_50_ for BK in female (**D**) and male (**E**) subjects with morbid obesity. pEC_50_ values for BK for each subject were obtained in HMAs contracted with the thromboxane analogue, U46619. Coefficients of determination and probability (*p*) values are indicated. Significant associations are highlighted in bold plus italic.

When data from obese subjects were segregated into female and male, no significant differences in BK-induced vasodilation were observed (Figure 3A–C). In addition, LDL-c/HDL-c ratio was able to predict the vasodilatory capacity of BK in both genders. A significant and inverse association of pEC50 with LDL-c/HDL-c ratio was obtained in arteries from obese women (Figure 3D) and men (Figure 3E).

### 3.7. Endothelial Vasodilation in Obese Subjects to BK Is Inversely Related to Vascular Expression of NADPH-Oxidase-2 (NOX2) and Superoxide Generation

**Figure 4:**
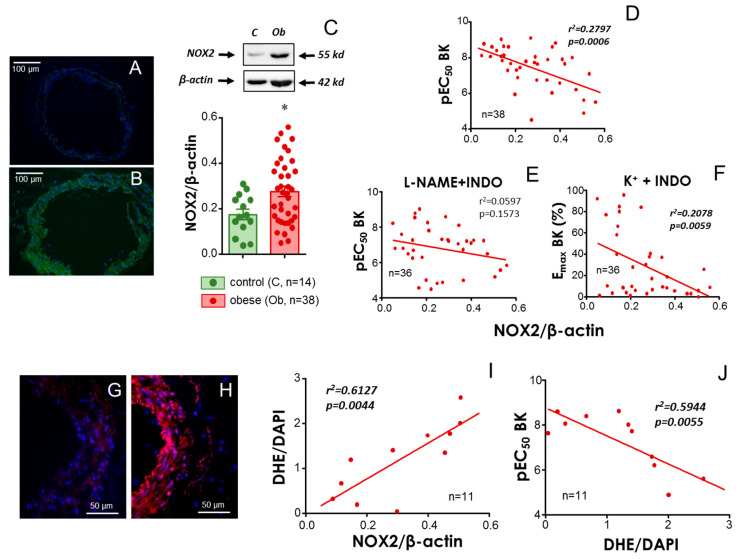
**The vascular expression of NADPH-oxidase-2 (NOX2) is related to increased oxidative stress and reduced endothelial vasodilation in obese subjects.** Panels (**A**,**B**) show representative immunofluorescence detection of NOX2 protein in sections of small mesenteric arteries from a control and an obese subject, respectively. Magnification ×200. High immunoreactivity (green fluorescence) is detected in the arterial wall of the obese subject. Nuclei are stained with DAPI (blue). Panel (**C**) shows representative immunoblots for the detection of NOX2 and corresponding β-actin in mesenteric artery homogenates from non-obese (control, **C**) and obese (Ob) subjects as well as the quantification of the expression assays. Data are expressed as the mean ± S.E.M. of NOX2 band intensities normalized by respective β-actin band intensities. * indicates *p* < 0.05 vs. control by the Mann–Whitney U test. Panels (**D**–**F**) show linear regressions of NOX2 expression (NOX2/β-actin ratio) versus pEC_50_ (**D**,**E**) or E_max_ for BK (**F**) in subjects with morbid obesity. BK-induced responses for each subject were obtained in HMAs contracted with the thromboxane analogue, U46619 (10–30 nM) (**D**,**E**), or 25–35 mM K^+^ (**F**). Data in panel (**D**) were obtained in HMAs without treatment, while HMAs were treated with indomethacin (INDO, 10 µM) plus N^G^-nitro-L-arginine methyl ester (L-NAME, 100 µM) in panel (**E**) and with INDO (10 µM) in panel (**F**). Panels (**G**,**H**) are representative images showing the detection of dihydroethidium (DHE) fluorescence (red) and DAPI staining (blue) in HMA sections from an obese subject with low (**G**) and high (H) DHE/DAPI fluorescence ratios. Magnification ×400. Panel (**I**) shows the linear regression of NOX2 expression in HMA homogenates versus superoxide generation determined by DHE/DAPI fluorescence in HMA sections from patients with morbid obesity, while panel (**J**) shows the linear regression of DHE/DAPI fluorescence versus pEC_50_ for BK in subjects with morbid obesity. pEC_50_ values for BK for each subject were obtained in HMAs contracted with U46619 and without further treatment. Coefficients of determination (r^2^) and probability (*p*) values are indicated in panels (**D**–**F**,**I**,**J**). Significant associations are highlighted in bold plus italic. *n* always indicates the number of subjects from whom the tissues were collected for determinations.

The expression of NADPH-oxidase-2 (NOX2) protein was detected by immunofluorescence assays and localized in the arterial wall from human small mesenteric arteries. Arterial sections from obese subjects apparently displayed higher immunoreactivity than those from control subjects (Figure 4A,B). For Western blot experiments, enough mesenteric vascular tissue was obtained from 38 obese subjects and 14 control subjects. Immunoblotting analyses on the expression of NOX2 revealed a significant increase in the expression of this superoxide-generating enzyme in vascular tissue from obese subjects with respect to control ones (Figure 4C). However, a heterogeneous level of NOX2 protein expression was observed among obese subjects. Interestingly, the level of NOX2 in vascular tissue in obese subjects significantly and negatively correlated with their respective vasodilatory efficacy to BK (Figure 4D). This association lost significance when EDH-mediated responses were specifically evaluated (Figure 4E) but remained significant when the NO-dependent component of endothelial vasodilation was isolated (Figure 4F). Since NOX2 activity results in superoxide production, we evaluated in situ ROS generation in vascular sections from some obese subjects (*n* = 11). There were clear differences in ROS generation in mesenteric arterial sections among obese subjects, as measured by DHE-derived fluorescence (Figure 4G,H). DHE intensity (red) normalized by blue DAPI-related fluorescence (nuclear staining) significantly and positively correlated with the expression of NOX2 in these vascular tissues (Figure 4I). Furthermore, fluorescence intensity from the DHE probe was, in turn, significantly and negatively correlated with BK-induced vasodilation (Figure 4J) in arteries from obese subjects, suggesting that increased expression of NOX2 compromises endothelial vasodilation in obese subjects by augmenting superoxide production in vascular tissue.

**Figure 5:**
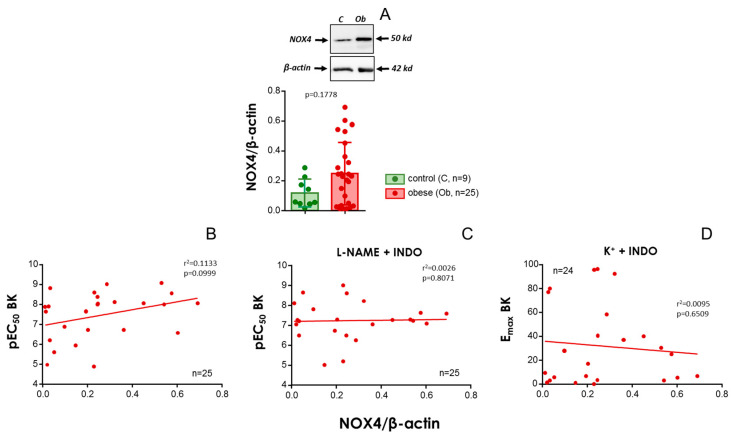
**The vascular expression of NADPH-oxidase-4 (NOX4) is not related to endothelial vasodilation in obese subjects.** Panel (**A**) shows representative immunoblots for the detection of NOX4 and corresponding β-actin in mesenteric artery homogenates from non-obese (control, C) and obese (Ob) subjects as well as the quantification of the expression assays. Data are expressed as the mean ± S.E.M. of NOX4 band intensities normalized by respective β-actin band intensities. *p* by the Mann–Whitney U test is indicated. Panels (**B**–**D**) show linear regressions of NOX4 expression (NOX4/β-actin ratio) versus pEC_50_ (**B**,**C**) or E_max_ for BK (**D**) in subjects with morbid obesity. BK-induced responses for each subject were obtained in HMAs contracted with the thromboxane analogue, U46619 (10–30 nM) (**B**,**C**), or 25–35 mM K^+^ (**D**). Data in panel (**B**) were obtained in HMAs without treatment, while HMAs were treated with indomethacin (INDO, 10 µM) plus N^G^-nitro-L-arginine methyl ester (L-NAME, 100 µM) in panel (**C**) and with INDO (10 µM) in panel (**D**). Coefficients of determination (r^2^) and probability (*p*) values are indicated in panels (**B**–**D**). *n* always indicates the number of subjects from whom the tissues were collected for determinations.

After evidencing the determinant role of NOX2 expression on endothelial vasodilation in arteries from obese subjects, we evaluated the expression of the isoform NOX4 in small mesenteric arteries from control (*n* = 9) and obese subjects (*n* = 25). A non-significant trend of higher expression of NOX4 was detected in vascular tissues from obese subjects (Figure 5A). Moreover, there were no significant associations of NOX4 expression with BK-induced responses (Figure 5B,D), indicating that, in contrast to NOX2, NOX4 does not have a determinant role in endothelial vasodilatory performance in small mesenteric arteries from obese subjects.

### 3.8. Elevated LDL-c/HDL-c and NOX2 Expression Could Additively Contribute to Defective Vasodilation in Obese Subjects

**Figure 6:**
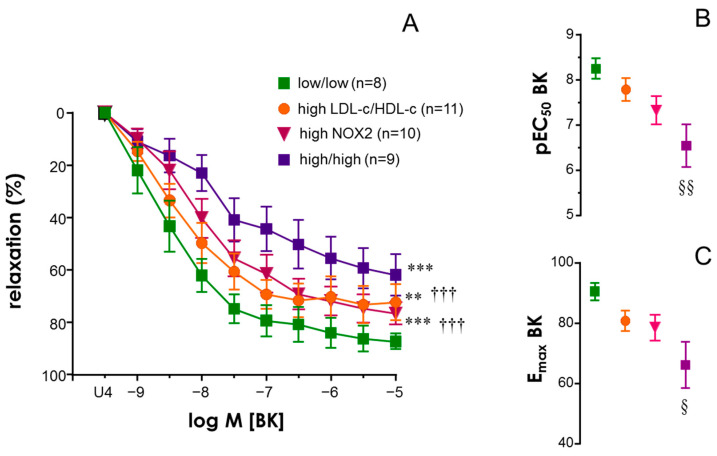
**The endothelial vasodilation of human mesenteric arteries (HMAs) from obese subjects is additively impaired by high LDL-c/HDL-c ratio and high vascular expression of NADPH-oxidase-2 (NOX2).** Panel (**A**) shows vasodilations induced by bradykinin (BK, 1 nM to 10 µM) in HMAs contracted with the thromboxane analogue, U46619 (10–30 nM), obtained from obese subjects with low LDL-c/HDL-c ratio (≤2) and low vascular expression of NOX2 (below median) (low/low), from obese subjects with high LDL-c/HDL-c ratio (above 2) but low NOX2 expression (high LDL-c/HDL-c), from obese subjects with high vascular expression of NOX2 (above median) but low LDL-c/HDL-c ratio (high NOX2) and from obese subjects with both high LDL-c/HDL-c and NOX2 expression (high/high). Data are expressed as the mean ± S.E.M. of the percentage of relaxation. *n* indicates the number of subjects from whom the tissues were collected for determinations. ** indicates *p* < 0.01, *** *p* < 0.001 vs. low/low, ††† *p* < 0.001 vs. high/high by two-factor ANOVA test corrected by Bonferroni’s test. Right panels show mean ± S.E.M. of pEC_50_ (**B**) and E_max_ (**C**) values for BK corresponding to each subject group. § *p* < 0.05, §§ *p* < 0.01 vs. low/low by Kruskal–Wallis followed by Dunn’s test.

Despite the fact that LDL-c/HDL-c ratio (even in the subset of 38 obese subjects with NOX2 determined, r = −0.4082, *p* = 0.0121) as well as NOX2 expression were independently associated with endothelial vasodilation decline in obese subjects, NOX2 expression was not significantly correlated to LDL-c/HDL-c in these subjects (r = 0.2898, *p* = 0.5890). This led us to think that LDL-c/HDL-c elevation and NOX2 upregulation were not different steps in the same pathway but they could additively contribute to compromising endothelial function in obese subjects. In this sense, we evaluated endothelial relaxation to BK in arteries from obese subjects in different conditions with respect to LDL-c/HDL-c ratio and the vascular expression of NOX2. This analysis revealed that those subjects without the elevation of plasma LDL-c/HDL-c (<2) and without the upregulation of NOX2 (below the median) exhibited BK-induced relaxation significantly improved with respect to subjects with elevated levels of LDL-c/HDL-c ratio and/or NOX2 vascular content (Figure 6A). However, those obese subjects presenting both LDL-c/HDL-c elevation and relative NOX2 high content displayed a significantly deeper impairment of BK-induced relaxation than those having only elevated LDL-c/HDL-c or NOX2 increase (Figure 6A), suggesting additive deleterious effects of both conditions on endothelial function in obese subjects. In fact, pEC_50_ and E_max_ for BK were significantly reduced in the group of obese subjects presenting the two parameters elevated (Figure 6B,C).

## 4. Discussion

The present results reveal important clues for the identification of obese subjects prone to displaying vascular alterations such as endothelial dysfunction, which can increase the risk for CVD. The presence of a disadvantageous lipid profile that is determined by high LDL-c/HDL-c ratio as well as an increase in vascular oxidative stress through NOX2 upregulation are associated with defective endothelial function in obese subjects as determined by BK-induced relaxation of mesenteric arteries. In fact, when high serum LDL-c/HDL-c ratio and high NOX2 vascular expression are present, an exacerbated impairment of endothelial function is detected in vessels from obese subjects.

Our functional evaluation of small vessels from morbidly obese subjects confirms the deleterious effect of obesity on endothelial function. This effect is produced in a population of persons with obesity that, in addition to having higher weight and BMI, and despite being younger, has a higher proportion of subjects with diabetes, insulin resistance, liver steatosis and sleep apnea. However, although these conditions probably account for the impact of obesity on endothelial function, they do not represent by themselves a determinant of endothelial dysfunction among morbidly obese subjects as evidenced by the lack of difference when segregating the subjects depending on the presence of such conditions, as shown in Table 2. In contrast, lipid profile and, specifically, LDL-c/HDL-c ratio significantly determine endothelial vasodilation as well as vasoconstriction in arteries from obese subjects. This result would be in agreement with evidence suggesting the role of LDL-c and HDL-c levels in influencing endothelial function in other kinds of populations. In this sense, in healthy subjects, HDL-c was reported to be predictive of endothelial function measured by plethysmography [36] and to be specifically associated with endothelial forearm vasodilation to methacholine [37]. In adult subjects, low acetylcholine- and BK-induced forearm endothelial vasodilation has been related to BMI, low HDL-c, high LDL-c and high total cholesterol/HDL-c ratio [38]. Moreover, high total cholesterol/HDL-c ratio has been shown to negatively determine FMD in both normotensive and hypertensive Japanese subjects [39]. In hyperlipemic men, low HDL-c is related to reduced FMD and increased expression of endothelial adhesion molecules [40]. Nevertheless, despite reducing LDL-c/HDL-c ratio, simvastatin did not improve endothelium-dependent vasodilation of the brachial artery determined by ultrasonography in hyperlipemic subjects [41], while estrogen therapy in postmenopausal women reduced LDL-c/HDL-c ratio and augmented brachial artery dilation [42]. Analysis of FMD in patients with elevated LDL-c or TG revealed no significant differences when compared to normolipemic subjects, while the ratio LDL-c/HDL-c was the only parameter predicting mild FMD impairment by multivariate analysis [43]. Our results support the role of LDL-c/HDL-c ratio in determining endothelial function in obesity by demonstrating a significant negative association between LDL-c/HDL-c and pEC_50_ for BK in mesenteric small arteries from obese subjects but also by showing a significant impairment of endothelial vasodilation in arteries from obese subjects with LDL-c/HDL-c >2 (more than two times more LDL-c than HDL-c) with respect to those obtained from obese subjects with LDL-c/HDL-c below this score. Moreover, the determinant role of LDL-c/HDL-c ratio was also observed for vasodilatory responses mediated by endothelial hyperpolarization (in arteries treated with L-NAME and indomethacin). The specific influence of LDL-c/HDL-c on NO-mediated responses (in arteries contracted with KCl and treated with indomethacin) was not so obvious because association between LDL-c/HDL-c and pEC_50_ for BK was not significant under these specific conditions, maybe because a significant number of subjects displayed almost absent vasodilation in this situation. However, subjects displaying the best NO-mediated vasodilation (highest tertile of E_max_ for BK) had significantly lower LDL-c/HDL-c ratios, suggesting the influence of LDL-c/HDL-c ratio also on NO-mediated vasodilation in mesenteric small arteries from obese subjects.

On the other hand, it should be noted that the role of LDL-c/HDL-c ratio in predicting microvascular endothelial function applies for morbidly obese men and women since the association of LDL-c/HDL-c with pEC_50_ for BK is significant in both cases. This points to an effect not depending on gender.

Trying to precisely delineate the determinants of endothelial dysfunction among obese subjects and considering the critical role of oxidative stress on obesity-related vascular dysfunction [44], we evaluated the vascular expression of NADPH-oxidase-2 (NOX2) in the study subjects. This was based on the key contribution of NOX activity to the rise of reactive oxygen species (ROS) and vascular pathophysiology [21,45] and on the fact that the upregulation of NOX2 has been proposed to participate in vascular alterations in different vascular beds from animal models of diet-induced obesity [46,47,48]. Moreover, NOX2 has been proposed as the main NOX isoform involved in aging-related endothelial dysfunction in human muscle feed arteries [49] as well as in the development of peripheral artery disease [50]. In fact, we evidenced an increased expression of NOX2 in mesenteric small arteries from obese subjects when compared to non-obese ones. Furthermore, among obese subjects, the vascular expression level of NOX2 significantly correlated to the level of vascular superoxide production and also negatively to vasodilatory capacity, suggesting that the level of NOX2-related generation of ROS probably determines endothelial function in the vasculature from obese subjects. This is in agreement with the reported increase in blood levels of soluble NOX2-derived peptide in obese and hypercholesterolemic children and the negative association of flow-mediated dilation and the levels of this peptide in children [51].

In addition to NOX2, the NOX4 isoform seems to have an important level of participation in vascular physiology and pathophysiology [24,25]. NOX4 has been proposed to contribute to vascular dysfunction in aging, diabetes and atherosclerosis [26,27,52], and it has shown to be upregulated in aortic tissue from rats with diet-induced obesity [53]. However, the role played by NOX4 is complex since protective actions on vascular function by NOX4 have been evidenced [28,29,54]. In fact, it has been shown to mediate the protective effects of physical activity against obesity-induced vascular dysfunction [55]. The explanation for these protective effects relies on the fact that NOX4 preferentially generates hydrogen peroxide instead of superoxide [56], promoting ROS-induced signaling such as hyperpolarization, eNOS activation or transient receptor potential vanilloid 4 (TRPV4) activation, all resulting in improved vasodilation [28,57,58,59]. The complexity of NOX4-mediated actions could be responsible for the lack of a net role of NOX4 expression on endothelial performance in small mesenteric arteries from obese subjects in the present study. Although the negative results with NOX4 could reinforce the preponderant role of NOX2 in conditioning endothelial vasodilation in mesenteric arteries from obese subjects, we cannot discard the contribution of additional NOX isoforms.

Despite the correlation of vasodilatory function in obese subjects with both LDL-c/HDL-c ratio and vascular NOX2 expression, no significant association was observed between these two parameters. In fact, the presence of both elevated LDL-c/HDL-c ratio and high NOX2 expression in obese subjects was related to a further impairment of endothelial vasodilation of mesenteric arteries when compared to those subjects presenting only elevated LDL-c/HDL-c ratio or high NOX2 expression. This would point to two mechanisms with additive impact on vascular function in obesity.

The ability of HDL-c to counteract the inhibitory effect of oxidized LDL-c on endothelium-dependent relaxation of rabbit aortic rings has been reported [60]. However, this capacity of HDL-c may be impaired when obtained from patients with abdominal obesity [61]. Moreover, the oxidation of HDL-c prevents this ability to abrogate the deleterious effect of oxidized LDL-c on endothelial function ex vivo [62]. In this sense, increased superoxide generation by upregulated NOX2 could favor HDL-c oxidation, compromising its protective capacities and exacerbating the impact of increased LDL-c/HDL-c ratio on endothelial function. Thus, the present results identify elevated LDL-c/HDL-c ratio and high expression of vascular NOX2 as indicators of poorer endothelial function in obese subjects. When these conditions are both present, the risk for endothelial impairment seems to increase, conferring a profile potentially prone to CVD development. It is interesting to highlight that while NOX2 upregulation mainly affected NO-mediated responses, elevated LDL-c/HDL-c was also associated with impaired EDH-mediated vasodilation. This would be in agreement with the observed impairment of both NO- and EDH-mediated vasodilatory responses in a rat model of bile duct ligation associated with elevated LDL-c and reduced HDL-c [63]. In this sense, LDL-c has been shown to inhibit hyperpolarization and inwardly rectifying potassium channel (K_IR_) activity in human aortic endothelial cells in vitro, while endothelial cells from hypercholesterolemic pigs with high LDL-c/HDL-c proportions displayed reduced hyperpolarization and K_IR_ activity [64].

The limitations of this study include the lack of intervention on the identified determinants for modifying their impact on endothelial function in obese subjects. Further research to overcome this limitation is guaranteed. The fact that the evaluation of NOX2 expression and ROS generation in vascular tissues could not be evaluated in the total number of subjects because of a lack of enough tissue amounts can be considered another limitation of the study. Finally, potential medications for different clinical conditions in the study population could influence the impact of such conditions on endothelial function, and this potential influence should be analyzed in future studies. Strengths of the study include the functional determination of endothelial function in an important number of human subjects and the approach to the determinants of endothelial function at several levels, finding a profile of high risk for endothelial impairment in persons with obesity.

## 5. Conclusions

Human obesity is related to an impairment of endothelial vasodilation, but endothelial function in obese subjects is variable and negatively associated with the serum LDL-c/HDL-c ratio and the vascular NOX2 expression. NOX2 upregulation was mainly related to a decline in NO-mediated responses, while elevated LDL-c/HDL-c was also associated with impaired EDH-mediated vasodilation. The presence of both elevated LDL-c/HDL-c ratio and high vascular expression of NOX2 would exacerbate the endothelial impairment in obesity and could indicate a deleterious profile for cardiovascular outcomes among obese subjects.

## Figures and Tables

**Table 1:** Characteristics of control and obese subjects.

Variable	Control	Obese	*p* Value
***n***	14	51	
**Age (years)**	57.4 ± 3.3	44.8 ± 1.5	***0.0012***
**Female sex (%)**	8 (57.1)	33 (64.7)	0.7559
**Weight (kg)**	71.5 ± 2.3	122.2 ± 3.5	***<0.0001***
**Height (m)**	1.63 ± 0.02	1.65 ± 0.01	0.3154
**BMI (kg/m^2^)**	26.8 ± 0.5	44.6 ± 1.1	***<0.0001***
**Diabetes (%)**	1 (7.1)	18 (35.3)	***0.0496***
**Dislipidemia (%)**	6 (42.9)	25 (49.0)	0.7681
**Hypertension (%)**	6 (42.9)	28 (54.9)	0.5489
**Sleep apnea (%)**	0 (0.0)	19 (37.2)	***0.0063***
**Liver steatosis (%)**	4 (28.6)	31 (60.8)	***0.0393***
**Smoking history (%)**	3 (21.4)	7 (13.7)	0.4384
**Coronary disease (%)**	1 (7.1)	1 (2.0)	0.3870
**Insulin (UI/mL)**	14.4 ± 3.1	24.1 ± 2.3	***0.0121***
**Glucose (mg/dL)**	93.6 ± 5.6	102.1 ± 5.3	0.2584
**HOMA-IR**	3.57 ± 0.86	6.33 ± 0.73	***0.0207***
**HbA_1C_ (%)**	5.61 ± 0.11	5.81 ± 0.16	0.9086
**Triglycerides (mg/dL)**	116.9 ± 17.8	119.4 ± 8.3	0.7019
**Total cholesterol (mg/dL)**	181.1 ± 8.4	177.9 ± 5.3	0.5093
**HDL-c (mg/dL)**	53.2 ± 5.0	52.1 ± 2.5	0.9528
**LDL-c (mg/dL)**	103.6 ± 10.7	104.0 ± 4.6	0.9843
**LDL-c/HDL-c**	2.25 ± 0.37	2.17 ± 0.13	0.7139
**AST (U/L)**	23.5 ± 3.1	21.8 ± 1.5	0.4167
**ALT (U/L)**	23.1 ± 3.9	28.6 ± 2.9	0.1508

ALT; alanine transaminase. AST; aspartate transaminase. BMI; body mass index. HDL-c; high-density lipoprotein cholesterol. HOMA-IR; homeostasis model assessment of insulin resistance. LDL-c; low-density lipoprotein cholesterol. TG; triglycerides. Numerical variables are expressed as mean ± S.E.M. and were compared by unpaired Mann–Whiney U-test, while categorical variables were compared by Fisher’s exact test. Significant associations are highlighted in bold plus italic.

**Table 2:** Relationships between analytical variables and endothelial vasodilation in study subjects.

	Control (*n* = 14)		Obese (*n* = 51)	
Variable	r	*p* Value	r	*p* Value
**Age (years)**	−0.0806	0.7845	0.0000	0.9629
**BMI (kg/m^2^)**	−0.2304	0.4280	0.1140	0.4248
**HOMA-IR**	−0.6111	***0.0202***	0.0001	0.9325
**HbA_1C_ (%)**	0.1615	0.5808	0.0574	0.6905
**Triglycerides (mg/dL)**	−0.2093	0.4728	−0.0510	0.7229
**Total cholesterol (mg/dL)**	0.1371	0.6397	−0.2278	0.1077
**HDL-c (mg/dL)**	0.0469	0.8746	0.2524	0.0739
**LDL-c (mg/dL)**	0.1487	0.6117	−0.4074	***0.0030***
**LDL-c/HDL-c**	−0.0387	0.8948	−0.5102	***0.0001***
**AST (U/L)**	−0.0900	0.7602	0.0922	0.5190
**ALT (U/L)**	−0.3507	0.2188	0.1221	0.3933

ALT; alanine transaminase. AST; aspartate transaminase. BMI; body mass index. HDL-c; high-density lipoprotein cholesterol. HOMA-IR; homeostasis model assessment of insulin resistance. LDL-c; low-density lipoprotein cholesterol. TG; triglycerides. Potential associations were evaluated by linear regression between analytical variables and the value of pEC_50_ (defined as the –log of the concentration needed to obtain 50% relaxation) for bradykinin in human mesenteric arteries as a measure of endothelial vasodilation in each subject. r indicates the correlation coefficient. Significant associations are highlighted in bold plus italic.

**Table 3:** Influence of comorbid conditions on endothelial function in obese subjects.

		Diabetes			Hypertension			Liver Steatosis			Sleep Apnea		
		Yes (18)	No (33)	*p*	Yes (28)	No (23)	*p*	Yes (31)	No (20)	*p*	Yes (19)	No (32)	*p*
**BK**	pEC_50_	7.53 ± 0.19	7.38 ± 0.24	0.6101	7.55 ± 0.22	7.30 ± 0.26	0.5386	7.64 ± 0.18	7.11 ± 0.31	0.1850	7.43 ± 0.21	7.45 ± 0.28	0.9545
	E_max_ (%)	78.1 ± 3.5	73.7 ± 3.5	0.6663	79.2 ± 2.8	70.6 ± 4.6	0.1773	76.7 ± 2.9	72.9 ± 5.0	0.6859	73.2 ± 4.2	76.4 ± 3.3	0.5686
**BK** **L-NAME +INDO**	pEC_50_	6.85 ± 0.31	6.92 ± 0.20	0.8196	7.00 ± 0.22	6.77 ± 0.26	0.4713	7.01 ± 0.23	6.71 ± 0.25	0.4475	6.84 ± 0.27	6.93 ± 0.22	0.6746
	E_max_ (%)	71.1 ± 5.3	70.2 ± 3.2	0.7900	73.2 ± 3.4	67.4 ± 4.6	0.4835	70.6 ± 3.5	70.3 ± 4.7	0.8730	68.8 ± 4.5	71.5 ± 3.6	0.5095
**BK** **KCl +INDO**	pEC_50_	5.26 ± 0.31	5.16 ± 0.20	0.9284	5.24 ± 0.25	5.14 ± 0.22	1.0000	5.40 ± 0.24	4.87 ± 0.20	0.3257	5.22 ± 0.27	5.18 ± 0.21	0.9909
	E_max_ (%)	29.2 ± 7.2	29.3 ± 5.6	0.4564	27.7 ± 6.2	31.0 ± 6.4	0.6170	34.1 ± 6.3	21.9 ± 5.3	0.4460	29.0 ± 6.9	29.4 ± 5.8	0.9922

pEC_50_ is the –log M of the concentration of bradykinin (BK) required for obtaining 50% relaxation, while E_max_ is the maximum percentage of relaxation induced by BK. L-NAME indicates the presence of the nitric oxide synthase inhibitor, N^G^-nitro-L-arginine methyl ester (100 µM), and INDO indicates the presence of the cyclooxygenase inhibitor, indomethacin (10 µM). KCl indicates that precontraction was achieved with 25–35 mM KCl instead of 10–30 nM of the thromboxane analogue, U46619. Data are expressed as mean ± S.E.M. The number of subjects for each condition is in parentheses. Data were compared by an unpaired Mann–Whiney U-test.

**Table 4:** Characteristics of obese subjects with the poorest endothelial function.

Variable	T2 + T3 pEC_50_ BK > 7.29	T1 pEC_50_ BK < 7.29	*p* Value
***n***	34	17	
**Age (years)**	45.2 ± 2.1	43.9 ± 2.0	0.5291
**Female sex (%)**	21 (61.8)	12 (70.6)	0.7568
**Weight (kg)**	124.6 ± 4.5	117.2 ± 5.3	0.4132
**Height (cm)**	166.1 ± 1.3	163.7 ± 2.0	0.2419
**BMI (kg/m^2^)**	45.1 ± 1.5	43.4 ± 1.2	0.9566
**Diabetes (%)**	12 (35.3)	6 (35.3)	1.0000
**Dislipidemia (%)**	17 (50.0)	8 (47.1)	1.0000
**Hypertension (%)**	20 (58.8)	8 (47.1)	0.5529
**Sleep apnea (%)**	13 (38.2)	6 (35.3)	1.0000
**Liver steatosis (%)**	24 (70.6)	7 (41.2)	0.0676
**Smoking history (%)**	3 (8.8)	4 (23.5)	0.2033
**Coronary disease (%)**	0 (0.0)	1 (5.9)	0.3333
**Insulin (UI/mL)**	23.8 ± 2.7	24.6 ± 4.1	0.6313
**Glucose (mg/dL)**	99.0 ± 4.0	108.2 ± 13.9	0.6451
**HOMA-IR**	5.95 ± 0.75	7.08 ± 1.62	0.7627
**HbA_1C_ (%)**	5.85 ± 0.23	5.74 ± 0.12	0.5145
**Triglycerides (mg/dL)**	116.4 ± 11.4	125.3 ± 10.2	0.2951
**Total cholesterol (mg/dL)**	172.1 ± 5.7	189.4 ± 11.0	0.1234
**HDL-c (mg/dL)**	55.2 ± 3.4	45.9 ± 2.8	***0.0293***
**LDL-c (mg/dL)**	95.7 ± 5.3	120.6 ± 7.3	***0.0066***
**LDL-c/HDL-c**	1.88 ± 0.13	2.77 ± 0.23	***0.0013***
**AST (U/L)**	22.7 ± 1.9	19.9 ± 2.5	0.2420
**ALT (U/L)**	30.9 ± 3.9	24.1 ± 3.4	0.1802

pEC_50_ BK is defined as the –log of the concentration of bradykinin needed to obtain 50% relaxation. ALT; alanine transaminase. AST; aspartate transaminase. BMI; body mass index. HDL-c; high-density lipoprotein cholesterol. HOMA-IR; homeostasis model assessment of insulin resistance. LDL-c; low-density lipoprotein cholesterol. TG; triglycerides. Numerical variables are expressed as mean ± S.E.M. and were compared by an unpaired Mann–Whiney U-test, while categorical variables were compared by Fisher’s exact test. Significant differences are highlighted in bold plus italic.

## Data Availability

All data are available upon request to corresponding author.

## Supplementary Materials

The following supporting information can be downloaded at: https://www.mdpi.com/article/10.3390/antiox13091139/s1, Figure S1: Low-density lipoprotein cholesterol (LDL-c)/high-density lipoprotein cholesterol (HDL-c) ratio is related to vasoconstriction in human mesenteric arteries (HMAs) from morbidly obese subjects; Table S1: Characteristics of obese subjects with the best response to bradykinin in KCl-contracted human mesenteric arteries.
