# Supplementary material for: LDL-c/HDL-c Ratio and NADPH-Oxidase-2-Derived Oxidative Stress as Main Determinants of Microvascular Endothelial Function in Morbidly Obese Subjects
Source: Antioxidants (Basel). 2024 Sep 20;13(9):1139. doi: 10.3390/antiox13091139 (PMC11444145; doi:10.3390/antiox13091139)
Supplement: Supplementary file 1 [file antioxidants-13-01139-s001.zip › antioxidants-3175227-supplementary.pdf]

Supplementary Table S1. Characteristics of obese subjects with the best response to bradykinin in KCl-contracted human mesenteric arteries

**Variable**	**T1+T2 (<30% E_{max})**	**T3 (>30% E_{max})**	**P value**
**n**	34	17	
**Age (years)**	44.2±1.8	45.5±2.8	0.6252
**Female sex (%)**	24 (68.6)	10 (58.8)	0.5439
**BMI (kg/m^2)**	44.7±1.5	45.6±1.6	0.2425
**Diabetes (%)**	12 (34.3)	6 (35.3)	1.0000
**Dislipidemia (%)**	17 (48.6)	6 (35.3)	0.3927
**Hypertension (%)**	20 (57.1)	9 (52.9)	1.0000
**Sleep apnea (%)**	12 (34.3)	7 (41.2)	0.7607
**Liver steatosis (%)**	21 (60.0)	11 (64.71)	0.7721
**Smoking history (%)**	4 (11.4)	3 (17.6)	0.6700
**Coronary disease (%)**	1 (2.9)	0 (0.0)	1.0000
**Insulin (mU/L)**	23.0±2.4	26.6±4.8	0.6885
**Glucose (mg/dL)**	99.3±6.9	106.8±7.4	0.1569
**HOMA-IR**	5.80±0.77	7.41±1.51	0.4650
**HbA_{1C} (%)**	5.69±0.09	6.06±0.43	0.9961
**Triglycerides (mg/dL)**	112.1±7.7	135.5±19.0	0.4081
**Total cholesterol (mg/dL)**	182.9±6.3	171.9±10.4	0.1252
**HDL-c (mg/dL)**	48.4±2.3	59.2±5.5	0.0730
**LDL-c (mg/dL)**	109.8±5.3	96.8±9.4	0.1882
**LDL-c/HDL-c**	2.43±0.16	1.79±0.21	***0.0179***
**AST (U/L)**	21.9±2.0	21.1±2.2	0.7752
**ALT (U/L)**	29.5±3.9	26.2±3.1	0.7978

ALT; alanine transaminase. AST; aspartate transaminase. BMI; body mass index. HDL-c; high-density lipoprotein cholesterol. HOMA-IR; homeostasis model assessment of insulin resistance. LDL-c; low-density lipoprotein cholesterol. TG; triglycerides. Numerical variables were compared by unpaired Mann-Whiney U-test while categorical variables where compared by Fisher’s exact test. Significant differences are highlighted in bold plus italic.
