# Supplementary material for: LDL-c/HDL-c Ratio and NADPH-Oxidase-2-Derived Oxidative Stress as Main Determinants of Microvascular Endothelial Function in Morbidly Obese Subjects
Source: Antioxidants (Basel). 2024 Sep 20;13(9):1139. doi: 10.3390/antiox13091139 (PMC11444145; doi:10.3390/antiox13091139)
Supplement: Supplementary file 1 [file antioxidants-13-01139-s001.zip › antioxidants-3175227-supplementary.pptx]

## Slide 1
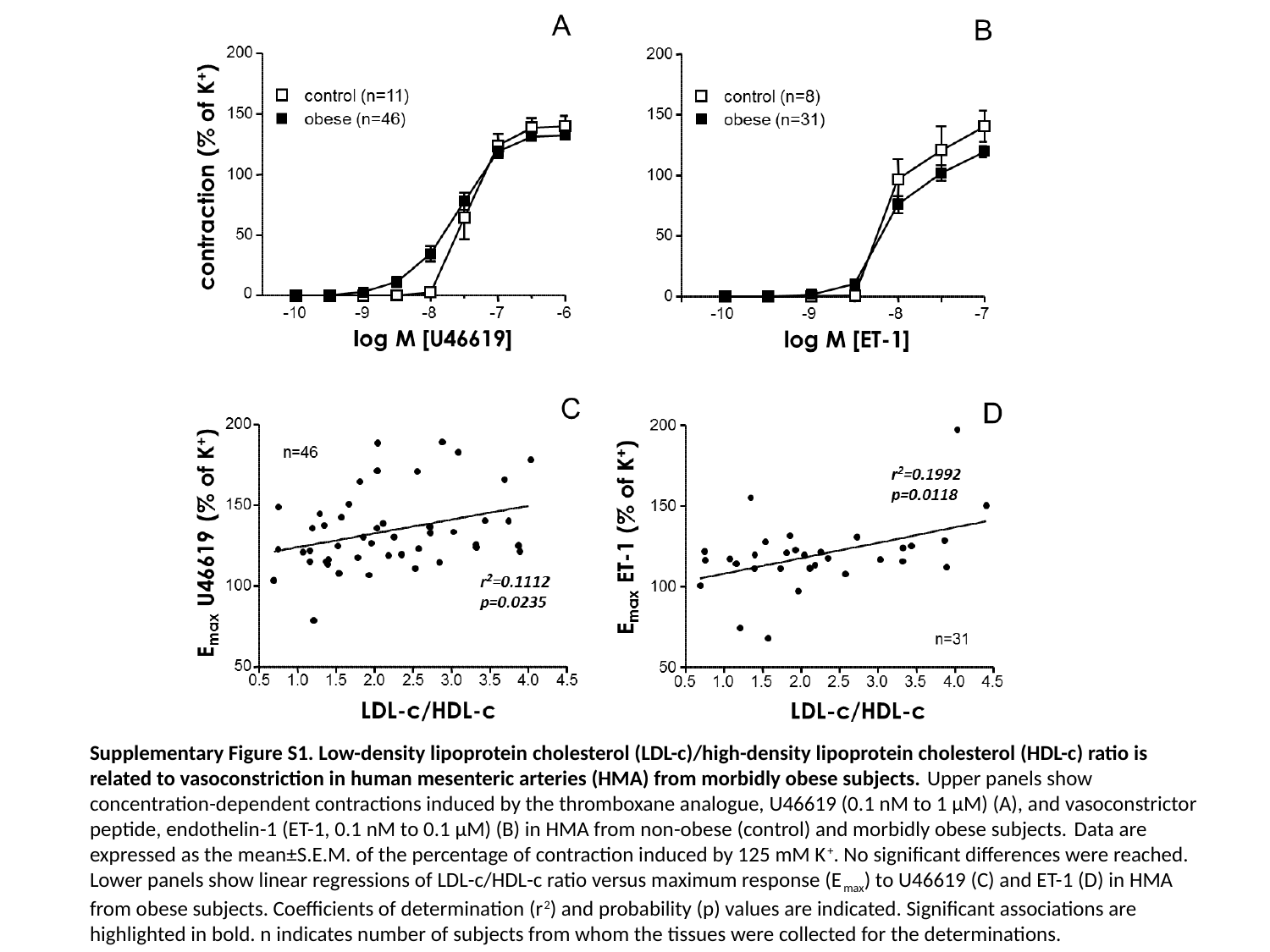

Supplementary Figure S1. Low-density lipoprotein cholesterol (LDL-c)/high-density lipoprotein cholesterol (HDL-c) ratio is related to vasoconstriction in human mesenteric arteries (HMA) from morbidly obese subjects. Upper panels show concentration-dependent contractions induced by the thromboxane analogue, U46619 (0.1 nM to 1 µM) (A), and vasoconstrictor peptide, endothelin-1 (ET-1, 0.1 nM to 0.1 µM) (B) in HMA from non-obese (control) and morbidly obese subjects. Data are expressed as the mean±S.E.M. of the percentage of contraction induced by 125 mM K+. No significant differences were reached. Lower panels show linear regressions of LDL-c/HDL-c ratio versus maximum response (Emax) to U46619 (C) and ET-1 (D) in HMA from obese subjects. Coefficients of determination (r2) and probability (p) values are indicated. Significant associations are highlighted in bold. n indicates number of subjects from whom the tissues were collected for the determinations.
